# Artificial Intelligence for Predicting HER2 Status of Gastric Cancer Based on Whole‐Slide Histopathology Images: A Retrospective Multicenter Study

**DOI:** 10.1002/advs.202408451

**Published:** 2025-01-10

**Authors:** Yuhan Liao, Xinhua Chen, Shupeng Hu, Bing Chen, Xinghua Zhuo, Hao Xu, Xiaojin Wu, Xiaofeng Zeng, Huimin Zeng, Donghui Zhang, Yunfei Zhi, Liang Zhao

**Affiliations:** ^1^ Department of Pathology Nanfang Hospital Southern Medical University Guangzhou 510515 China; ^2^ Department of Pathology Guangdong Province Key Laboratory of Molecular Tumor Pathology School of Basic Medical Sciences Southern Medical University Guangzhou 510515 China; ^3^ Department of General Surgery Nanfang Hospital Southern Medical University Guangzhou 510515 China; ^4^ School of Computer Science University of Manchester Manchester M13 9PL UK; ^5^ Department of Pathology Shunde Hospital of Southern Medical University (The First People's Hospital of Shunde) Foshan Guangdong 528399 China; ^6^ Department of Pathology Affiliated Cancer Hospital and Institute of Guangzhou Medical University Guangzhou 510095 China; ^7^ Department of Gastroenterology Chinese Academy of Medical Sciences Peking Union Medical College Hospital Beijing 100730 China

**Keywords:** deep learning model, gastric cancer, human epidermal growth factor receptor 2, whole slide images

## Abstract

Human epidermal growth factor receptor 2 (HER2) positive gastric cancer (GC) shows a robust response to the combined therapy based HER2‐targeted therapy. The application of these therapies is highly dependent on the evaluation of tumor HER2 status. However, there are many risks and challenges in HER2 assessment in GC. Therefore, an economically viable and readily available instrument is requisite for distinguishing HER2 status among patients diagnosed with GC. The study has innovatively developed a deep learning model, HER2Net, which can predict the HER2 status by quantitatively calculating the proportion of HER2 high‐expression regions. The HER2Net is trained on an internal training set derived from 531 hematoxylin & eosin (H&E) whole slide images (WSI) of 520 patients. Subsequently, the performance of HER2Net is validated on an internal test set from 115 H&E WSI of 111 patients and an external multi‐center test set from 102 H&E WSI of 101 patients. The HER2Net achieves an accuracy of 0.9043 on the internal test set, and an accuracy of 0.8922 on an external test set from multiple institutes. This discovery indicates that the HER2Net can potentially offer a novel methodology for the identification of HER2‐positive GC.

## Graphical abstract

This study introduces HER2Net, a deep learning model for identifying human epidermal growth factor receptor 2 (HER2) positive gastric cancer (GC) using hematoxylin and eosin (H&E) whole slide images (WSI). HER2Net quantifies HER2 high‐expression regions, potentially replacing current clinical assessment methods. It offers an accessible, cost‐effective solution with high accuracy, aiding in selecting GC patients for HER2‐targeted therapy.

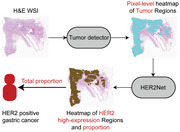

## 1 Introduction

GC represents a significant global health concern, predominantly in East Asian nations.^[^ ^1^, ^2^ ^]^ On a global scale, over one million instances were reported, culminating in upward of 7 68 000 fatalities in 2020. This positions GC as the fifth most prevalent cancer worldwide and the fourth leading contributor to cancer‐related mortalities.^[^ ^3^, ^4^ ^]^ The recombinant antibody for HER2 represents the sole targeted pharmaceutical intervention that has demonstrated efficacy in the treatment of patients with advanced GC.^[^ ^5^, ^6^ ^]^ The oncogene HER2, also referred to as ERBB2, undergoes amplification and the corresponding protein HER2 is high‐expressed in ≈17–20% of patients diagnosed with GCs. Individuals exhibiting a high‐expression of HER2 in GC derive therapeutic advantages from the administration of the anti‐HER2 antibody, Trastuzumab.^[^ ^7^ ^]^ Trastuzumab represents a humanized monoclonal antibody that specifically targets the HER2 receptor, consequently inhibiting the activation of downstream signals and inducing a phenomenon known as antibody‐dependent cellular toxicity. In clinical practice, the general process for the histopathological diagnosis of GC is to evaluate the samples obtained by hematoxylin & eosin (H&E) staining, and then, in accordance with the HER2 testing guidelines proposed by the College of American Pathologists, the American Society for Clinical Pathology, and ASCO,^[^ ^8^ ^]^ the NCCN Guidelines advocate for the utilization of immunohistochemistry (IHC) and, if necessitated, in situ hybridization (ISH) methodologies for the evaluation of HER2 status in GC.

However, there are many risks and challenges in HER2 assessment in GC. Factors such as inconsistent evaluation methods of biopsy samples and surgical samples, lax quality control of sample site and quantity, interobserver agreements and unsatisfactory diagnostic performances of IHC, and low fluorescence in situ hybridization (FISH) reinspection rate all affect the assessment of HER2 status.^[^ ^9^, ^10^, ^11^ ^]^

An increasing body of evidence suggests that deep learning can play a significant role in clinical diagnostic and prognostic tasks.^[^ ^12^ ^]^ Specifically, deep learning has been efficaciously utilized to distinguish diverse cancer subtypes and molecular characteristics on tissue pathology slides stained with H&E.^[^ ^13^, ^14^ ^]^ However, there have been few published studies in the field of GC. Deep learning models were used to predict microsatellite instability tumors (MSI) with the highest area under the receiver operating characteristic curve (AUROC) of 0.81^[^ ^15^ ^]^ and Epstein‐Barr virus (EBV) GC with the highest AUROC of 0.941,^[^ ^16^ ^]^ respectively. Both of these studies used over 1000 H&E WSIs from multiple centers for training and prediction to demonstrate the credibility of their conclusions. Recently, a study proposed a deep learning model that can simultaneously predict multiple subtypes, including EBV+, MSI, tumor mutational burden, and programmed cell death ligand 1, with the best performance ranging from AUROC 0.8685 to 0.9461.^[^ ^17^ ^]^ However, this study only tested on a single‐center test set with only 118 H&E WSI. In our review of related work in the past 5 years, we also found three other studies that used H&E WSI to predict GC subtypes.^[^ ^18^, ^19^, ^20^ ^]^ To the best of our understanding, this study represents the inaugural attempt at employing a deep learning model for the prediction of HER2 status in gastric cancer by utilizing H&E WSI, and we will also analyze the relationship between the model results and H&E WSI morphological features.

## 2 Results

### 2.1 Patients Cohorts

**Figure 1:**
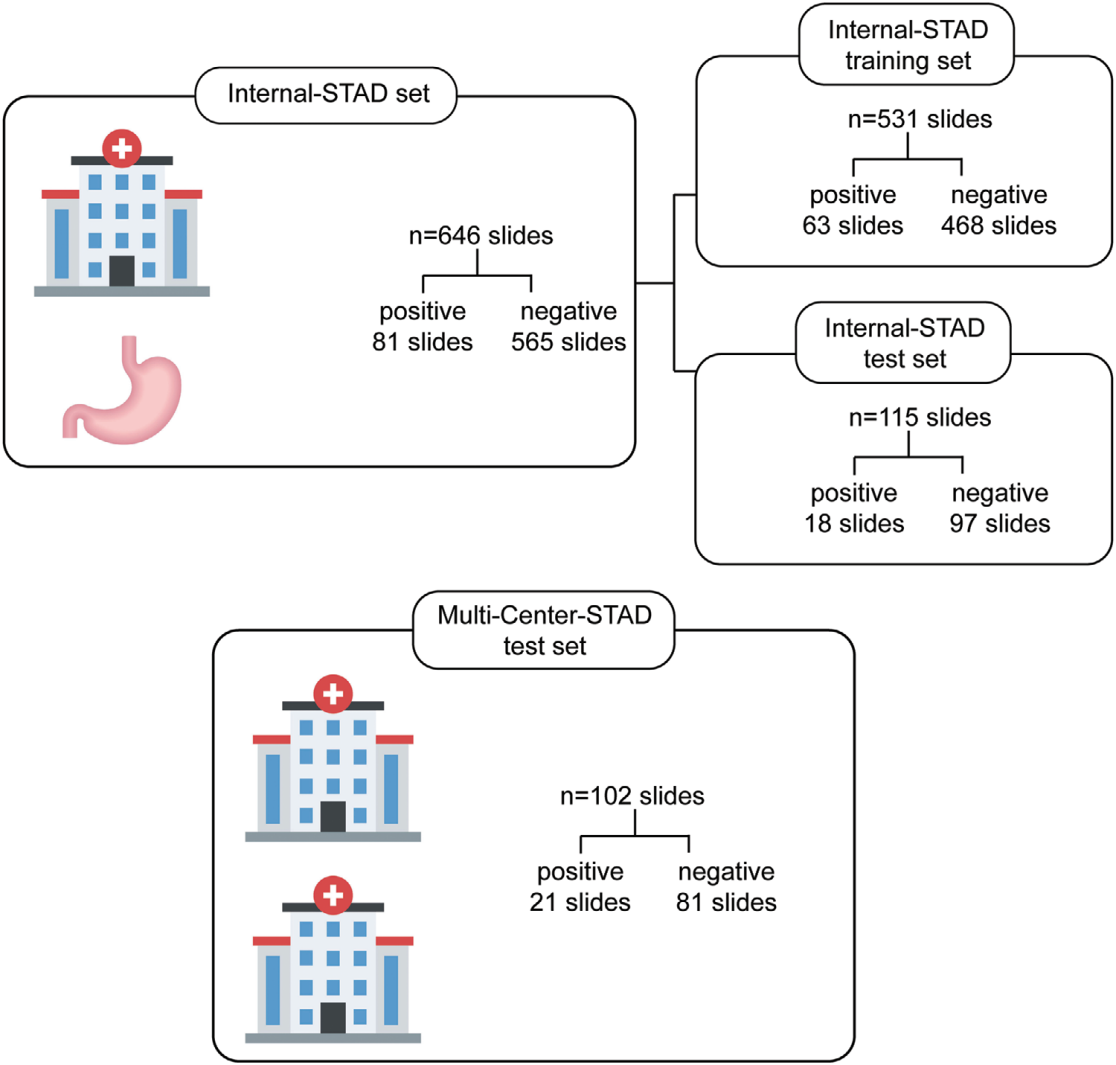
Brief usage summary of the internal set and the external set. The Internal‐STAD set was divided into an Internal‐STAD training set and an Internal‐STAD test set in a ratio of 82% to 18%. The Internal‐STAD training set was used for training the model using a stratified fivefold cross‐validation approach, while the internal testing set was solely used for testing. The proportion of positive samples in the internal training set was 11.86%, while in the internal testing set it was 15.65%. The Multi‐Center‐STAD test set, on the other hand, was an external test set used exclusively for testing, with a proportion of positive samples of 20.59%.

This study used both internal datasets and external multi‐center datasets as shown in **Figure** **1** and **Table** **1**. The Internal‐Stomach Adenocarcinoma (STAD) dataset served as the internal dataset and was divided into a training set for developing HER2Net and a testing set for evaluation. The training set consists of 531 H&E WSI from 63 HER2‐positive patients (63 slides) and 457 HER2‐negative patients (468 slides), accounting for 82.2% of the Internal‐STAD dataset. The testing set consists of 115 H&E WSI from 17 HER2‐positive patients (18 slides) and 94 HER2‐negative patients (97 slides), accounting for 17.8% of the Internal‐STAD dataset. To ensure better generalization of the trained model, we employed stratified 5‐fold cross‐validation at WSI‐level on the training set to ensure consistent proportions of HER2‐positive samples in each fold. Furthermore, the Internal‐STAD testing set has a similar proportion of positive samples (15.38%) to the Internal‐STAD training set (11.86%). The Multi‐Center‐STAD dataset served as the external dataset and is solely used for testing. It includes 102 H&E WSI from 20 HER2‐positive patients (21 slides) and 81 HER2‐negative patients (81 slides). In all the aforementioned datasets, each H&E WSI is paired with a corresponding IHC WSI.

**Table 1:** Clinical information of study participants.

Clinicopathological features		Internal‐STAD	Multicenter‐STAD
Patients		632	101
Slides		646	102
HER2 status	HER2 positive slides	81 (12.5%)	21 (20.6%)
	HER2 negative slides	565 (87.5%)	81 (79.4%)
Age (Mean age ± SD)		57.647 ± 11.950	56.843 ± 12.020
Gender	Male	418 (64.7%)	70 (68.6%)
	Female	228 (35.3%)	32 (31.4%)
Differentiation	Poor differentiation	509 (78.8%)	78 (76.5%)
	Middle and well differentiation	137 (21.2%)	24 (23.5%)

### 2.2 Performances of Pixel‐Level Tumor Detector

In clinical pathological diagnosis, the determination of HER2 status is only related to the histopathologic features of the tumor portion of the H&E WSI, and is unrelated to non‐tumor and background areas. Therefore, we need to train a pixel‐level tumor detector to identify the tumor pixels in the H&E WSI, and non‐tumor and background regions will not be used for model training and inference. Based on the experiment results, our pixel‐level tumor detector achieved the best average performance with an Mean Intersection over Union(MIoU) of 0.8606 on the Internal‐STAD test set and an MIoU of 0.8207 on the Multi‐Center‐STAD test set.

### 2.3 Performance of HER2Net

**Figure 2:**
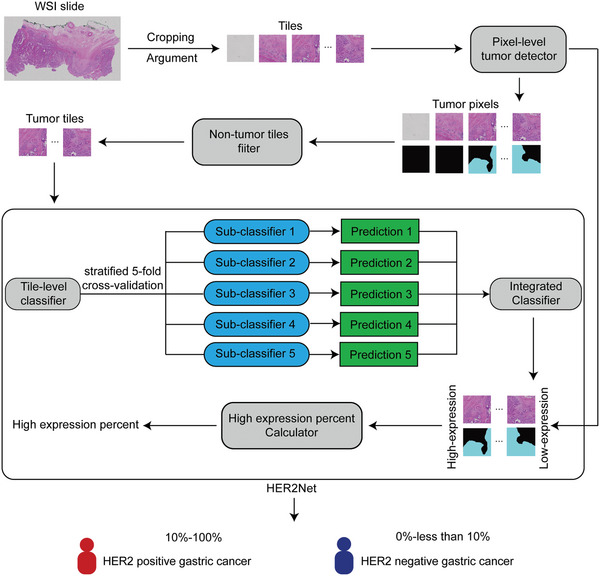
The workflow of HER2Net. Every H&E WSI was cropped into non‐overlapping 512‐by‐512 resolution H&E tiles of ×4 magnification. Afterward, data augmentation strategies were applied to the H&E tiles. Only tumor files where the percentage of tumor pixels predicted by the pixel‐level tumor detector is more than 10%, and the saturation is >10, were fed to HER2Net to achieve the WSI‐level high‐expression percentage. The H&E WSI was determined as HER2 positive by HER2Net when high‐expression percentage was greater than or equal to 10%.

HER2Net consists of three parts shown in **Figure** **2**: tile‐level classifier, integrated classifier, and high‐expression percent calculator. The tile‐level classifier was divided into five sub‐classifiers based on stratified 5‐fold cross‐validation, and each sub‐classifier predicted the classification of the same H&E tile. Therefore, a single H&E tile can yield five prediction results from five sub‐classifiers. In the stratified 5‐fold cross‐validation of the internal training set, the best average performance of the tile‐level classifier was an AUROC of 0.9379 displayed in Table S2 (Supporting Information). The integrated classifier was trained to learn the final classification prediction by combining the classification results obtained from the previous five tile‐level sub‐classifiers. The best performance of the integrated classifier on Multi‐Center‐STAD test set was an AUROC of 0.9769 and the sensitivity was 0.9373 displayed in Table S3 (Supporting Information). The high‐expression proportion calculator was used to calculate the proportion of high‐expression that could distinguish the HER2 status of H&E WSI. Basically, the best performance of HER2Net, composed of a combination of SegNet, ResNet50, and RF models, was an accuracy of 0.9043 on the Internal‐STAD test set and 0.8922 on the Multi‐Center‐STAD test set. All metric data is presented in Table S4 (Supporting Information) and the confusion matrix is shown in Figure S2 (Supporting Information).

### 2.4 Misdiagnosis from HER2Net

**Figure 3:**
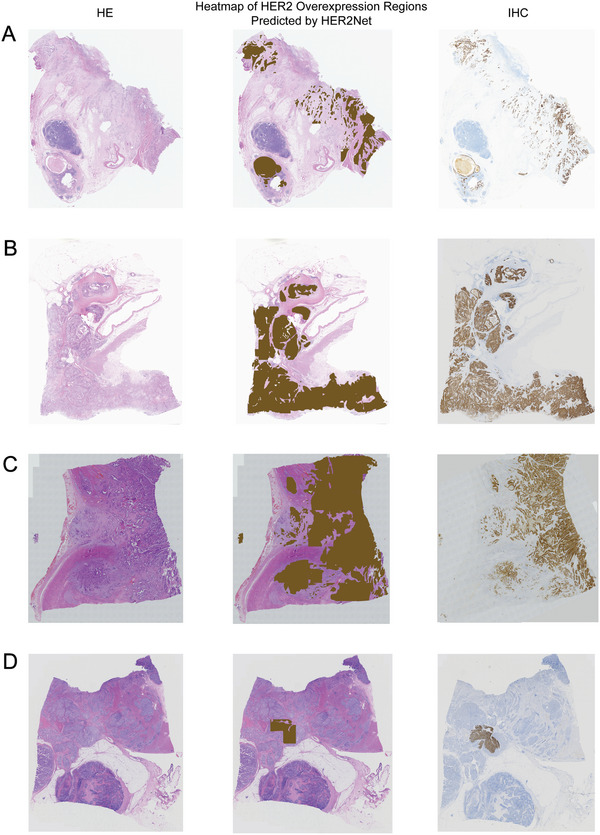
Successful cases predicted by HER2Net. Patients A, B, C, and D were all from the Multi‐Center‐STAD test set. Among them, A, B, and C were HER2‐positive, while D was HER2‐negative. The left column in the figure showed the H&E WSI of the patients, the right column showed the IHC WSI, and the middle column showed the heatmap predicted by HER2Net, which overlaid on the H&E WSI. The brown regions in the heatmap represented high‐expression areas consisting of tumor regions of H&E tiles classified as strong by HER2Net.

**Figure 4:**
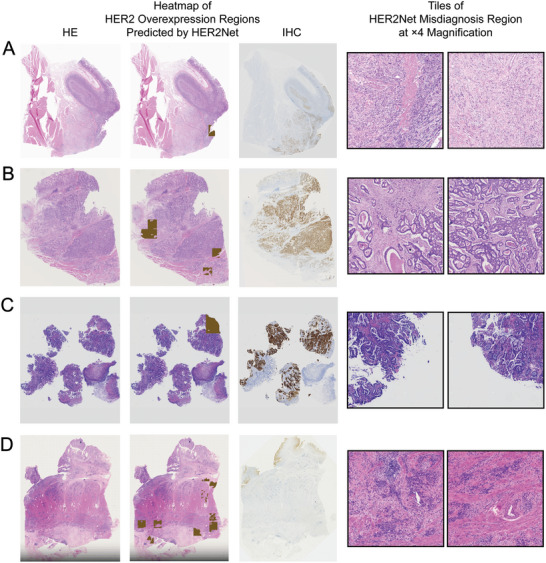
Unsuccessful cases predicted by HER2Net. Patients A, B, C, and D were all patients from the Multi‐Center‐STAD test set. Among them, A, B, and C were HER2‐positive but were incorrectly predicted as negative by HER2Net, while D was HER2‐negative but was incorrectly predicted as positive by HER2Net. The first column (counted from left to right) in the figure showed the H&E WSI of the patients, the third column showed the IHC WSI, and the second column showed the heatmap of HER2 high‐expression regions predicted by HER2Net, which overlaid on the H&E WSI. The brown areas in the heatmap represented high‐expression regions consisting of regions of H&E tiles classified as strong by HER2Net. In the heatmaps misdiagnosed as HER2‐negative by HER2Net (A, B, and C), the primary morphological characteristics of the brown areas that were not correctly identified, include poor differentiation of cancerous glandular ducts presenting as tubular, sieve‐like, cord‐like or scattered infiltration, an increased nucleocytoplasmic ratio, deeply stained nuclei, fibrosis of the interstitium, and scattered lymphocytic infiltration. In the heatmaps misdiagnosed as HER2 positive by HER2Net, the primary morphological characteristics of the erroneously identified brown regions are that the cancer cells are arranged in a linear pattern or focally clustered, with a significant increase in the nucleocytoplasmic ratio, scant cytoplasm, and deeply stained nuclei. The morphological features of the misdiagnosed regions by HER2Net are presented in the tiles with a magnification of ×4 in the fourth column.

In order to better analyze the relationship between the performance of HER2Net and histopathological features, we collected the morphological features of all H&E WSI, and employed the Chi‐square test to analyze the significance of the differences between the samples diagnosed correctly and incorrectly by HER2Net. The number of morphological features on different datasets were shown in **Table** **2**. In order to scrutinize the morphological features of the erroneously diagnosed cases by HER2Net, a comparative analysis in was conducted between the attributes of false positive instances and true negative instances, as well as between the attributes of false‐negative instances and true‐positive instances. On the Internal‐STAD test set comprising 115 slides, HER2Net misdiagnosed 11 slides, with 9 HER2‐negative cases erroneously predicted as HER2‐positive and 2 HER2‐positive cases inaccurately predicted as HER2‐negative. In **Table** **3**, compared to true negative cases, false positive cases were more likely to have the presence of adenoid differentiation (*P* = 0.014). On the Multi‐Center‐STAD test set consisting of 102 slides, HER2Net misdiagnosed 11 slides, where 8 HER2‐negative cases were incorrectly predicted as HER2‐positive and 3 HER2‐positive cases were wrongly predicted as HER2‐negative. In **Table** **4**, compared to true negative cases, false positive cases were more likely to have the presence of papillary differentiation (*P* = 0.018). **Figures** **3** and **4** presented some WSIs of successful and unsuccessful cases predicted by HER2Net respectively. In the heatmaps misdiagnosed as HER2‐negative by HER2Net, the primary morphological characteristics of the brown areas that were not correctly identified, include poor differentiation of cancerous glandular ducts presenting as tubular, sieve‐like, cord‐like, or scattered infiltration, an increased nucleocytoplasmic ratio, deeply stained nuclei, fibrosis of the interstitium and scattered lymphocytic infiltration. In the heatmaps misdiagnosed as HER2 positive by HER2Net, the primary morphological characteristics of the erroneously identified brown regions are that the cancer cells are arranged in a linear pattern or focally clustered, with a significant increase in the nucleocytoplasmic ratio, scant cytoplasm, and deeply stained nuclei.

**Table 2:** Number of morphological features on different datasets.

Morphological features		Internal‐STAD training set (n = 531)	Internal‐STAD test set (n = 115)	Multi‐Center‐STAD test set (n = 102)
Tertiary lymphoid structure	Presence	388 (73.07%)	82 (71.30%)	65 (63.73%)
	Absence	143 (26.93%)	33 (28.70%)	37 (36.27%)
Mucinous differentiation	Presence	110 (20.72%)	26 (22.61%)	12 (11.76%)
	Absence	421 (79.28%)	89 (77.39%)	90 (88.24%)
Adenoid differentiation	Presence	312 (58.76%)	60 (52.17%)	65 (63.73%)
	Absence	219 (41.24%)	55 (47.83%)	37 (36.27%)
Papillary differentiation	Presence	47 (8.85%)	7 (6.09%)	11 (10.78%)
	Absence	484 (91.15%)	108 (93.91%)	91 (89.12%)
Signet‐ring cell	Presence	185 (34.84%)	37 (32.17%)	28 (27.45%)
	Absence	346 (65.16%)	78 (67.83%)	74 (72.55%)
Poor differentiation	Presence	408 (76.84%)	89 (77.39%)	71 (69.61%)
	Absence	123 (23.16%)	26 (22.61%)	31 (30.39%)

**Table 3:** The comparison of HER2Net misdiagnosed and correctly diagnosed cases on Internal‐STAD test set.

Clinicopathological features		HER2‐positive (n = 18)			HER2‐negative (n = 97)		
		False negative (n = 2)	True positive (n = 16)	P	False positive (n = 9)	True negative (n = 88)	P
Gender	Mean age	55	59	–	58.22	55.24	–
	Male	2 (100%)	12 (75%)	1	7 (77.78%)	50 (56.82%)	0.389
	Female	0 (0%)	4 (25%)		2 (22.22%)	38 (43.18%)	
Tertiary lymphoid structure	Presence	1 (50%)	8 (50%)	1	8 (88.89%)	65 (73.86%)	0.556
	Absence	1 (50%)	8 (50%)		1 (11.11%)	23 (26.14%)	
Mucinous differentiation	Presence	1 (50%)	1 (6.25%)	0.507	2 (22.22%)	22 (25%)	1
	Absence	1 (50%)	15 (93.75%)		7 (77.78%)	66 (75%)	
Adenoid differentiation	Presence	2 (100%)	14 (87.5%)	1	7 (77.78%)	27 (30.68%)	0.014
	Absence	0 (0%)	2 (12.5%)		2 (22.22%)	61 (69.32%)	
Papillary differentiation	Presence	0 (0%)	3 (18.75%)	1	1 (11.11%)	3 (3.41%)	0.821
	Absence	2 (100%)	13 (81.25%)		8 (88.89%)	85 (96.59%)	
Signet‐ring cell	Presence	0 (0%)	2 (12.5%)	1	2 (22.22%)	33 (37.5%)	0.586
	Absence	2 (100%)	14 (87.5%)		7 (77.78%)	55 (62.5%)	
Poor differentiation	Presence	0 (0%)	6 (37.5%)	0.791	7 (77.78%)	76 (86.36%)	0.841
	Absence	2 (100%)	10 (62.5%)		2 (22.22%)	12 (13.64%)	

**Note**: The features of misdiagnosed cases were compared with those of correctly diagnosed cases with the Chi‐square test with 95% confidence level.

**Table 4:** The comparison of HER2Net misdiagnosed and correctly diagnosed cases on Multi‐Center‐STAD test set.

Clinicopathological features		HER2‐positive (n = 21)			HER2‐negative (n = 81)		
		False negative (n = 3)	True positive (n = 18)	P	False positive (n = 8)	True negative (n = 73)	P
Gender	Mean age	55	66.5	–	59.875	54.21	–
	Male	2 (66.67%)	14 (77.78%)	1	5 (62.5%)	49 (67.12%)	1
	Female	1 (33.33%)	4 (12.22%)		3 (37.5%)	24 (22.88%)	
Tertiary lymphoid structure	Presence	1 (33.33%)	6 (33.33%)	1	4 (50%)	54 (73.97%)	0.310
	Absence	2 (66.67%)	12 (66.67%)		4 (50%)	19 (26.03%)	
Mucinous differentiation	Presence	1 (33.33%)	0 (0%)	0.296	2 (25%)	9 (12.33%)	0.653
	Absence	2 (66.67%)	18 (100%)		6 (75%)	64 (87.67%)	
Adenoid differentiation	Presence	2 (66.67%)	17 (94.44%)	0.649	6 (75%)	40 (54.79%)	0.472
	Absence	1 (33.33%)	1 (5.56%)		2 (25%)	33 (45.21%)	
Papillary differentiation	Presence	0 (0%)	8 (44.44%)	0.409	2 (25%)	1 (1.37%)	0.018
	Absence	3 (100%)	10 (55.56%)		6 (75%)	72 (98.63%)	
Signet‐ring cell	Presence	0 (0%)	0 (0%)	NA	1 (12.5%)	27 (37.00%)	0.321
	Absence	3 (100%)	18 (100%)		7 (87.5%)	46 (63.00%)	
Poor differentiation	Presence	1 (33.33%)	6 (33.33%)	1	4 (50%)	60 (82.19%)	0.096
	Absence	2 (100%)	10 (62.5%)		2 (22.22%)	12 (13.64%)	

**Note**: The features of misdiagnosed cases were compared with those of correctly diagnosed cases with the Chi‐square test with 95% confidence level. NA, in the input observations, if any set of data is entirely zero, it is impossible to conduct a Chi‐square test.

## 3 Discussion

In the field of deep learning research on tumors based on H&E WSI pathology, no pixel‐level models suitable for clinical diagnosis have been developed for tumor detection.^[^ ^21^ ^]^ Existing research typically employs two methods: one is manual annotation of tumor regions on each H&E WSI by pathologists,^[^ ^18^ ^]^ a method that, while accurate, is labor‐intensive and difficult to adapt to clinical needs; the other is training a binary model at the H&E tile‐level,^[^ ^16^ ^]^ marking tiles with over 50% tumor tissue area as tumor images, and the rest as non‐tumor images. However, this method overlooks the variation in tumor area across different tiles, and its accuracy can be affected by the complexity of the tissue space and background in the tiles. Therefore, our research innovatively proposes the use of a pixel‐level tumor detection model for AI tumor research based on H&E WSI, with the aim of improving model accuracy and achieving better AI clinical application results.

The interpretation of HER2 status in the clinic depends on its intensity and proportion. To date, there are no deep learning methods that can be referenced, so we propose an innovative semi‐quantitative model—HER2Net. HER2Net can quantitatively calculate the proportion of high‐expression areas of HER2, and determine the HER2 status of patients based on the proportion threshold in clinical diagnosis. In contrast, previous related studies' deep learning methods mainly focused on the binary classification prediction of cancer subtypes qualitatively,^[^ ^15^, ^16^, ^17^, ^18^, ^19^, ^20^ ^]^ the main limitation of which is the inability to provide the specific proportion of high‐expression areas, making it difficult to meet the need for precise quantitative information in clinical diagnosis. The deep learning method proposed in our study not only overcomes this limitation, but also provides a reference for semi‐quantitative analysis of other immunohistochemical indicators, and can be broadly extended to the interpretation of other indicators.

The contributions of this study to clinical practice primarily encompass two aspects. First, the high cost of HER2 assessment and the accessibility of FISH hinder the clinical testing of many patients.^[^ ^9^, ^10^, ^11^ ^]^ Our proposed HER2Net, which directly diagnoses the HER2 status based on H&E WSI, has the potential to replace current clinical HER2 assessment methods, or serve as supportive evidence prior to clinical HER2 evaluation. Second, the challenging interpretation of IHC WSI leads to poor consistency in pathologists' reading results. Our proposed HER2Net can assist pathologists in clinical interpretation by predicting the high‐expression areas of HER2 and calculating its area ratio at the pixel level.

A sufficient volume of data can significantly mitigate the potential generalization issues of deep learning models. To the best of our knowledge, the number of HER2 patients and their corresponding H&E WSIs collected in this study, particularly the number of HER2‐positive cases, may be the largest to date in gastric cancer HER2 deep learning research based on H&E WSIs. More experimental data can assist us in training models with enhanced generalization capabilities and robustness. We were inspired and employed a 5‐fold cross‐validation approach to further enhance the generalization capability of the model.^[^ ^16^ ^]^ However, in clinical diagnosis, the number of HER2‐positive patients is significantly less than that of HER2‐negative patients, leading to an imbalance in the proportion of HER2‐positive and negative samples in the collected HER2 case sections. Therefore, we additionally employed a stratified sampling method to ensure that the proportion of HER2‐positive samples in each fold is as consistent as possible. The results indicated that this approach can further enhance the model's generalization ability based on 5‐fold cross‐validation.

Processing methods for H&E WSI in gastric cancer deep learning research primarily include: 1) cropping; 2) resizing; 3) color normalization; and 4) data augmentation. H&E WSIs are often high‐resolution images that cannot be directly used in deep learning models. Therefore, it is a common data processing practice to crop H&E WSIs into a certain number of H&E tiles and train models at the tile level. When cropping H&E tiles, we ensure that they do not overlap, as HER2Net's pixel‐level predictions and calculations do not allow for pixel overlap. The dimensions of the cropped H&E tiles are often inconsistent. A common approach is to resize all H&E tiles to a uniform size; however, this can lead to pixel distortion, which is detrimental to the training of our pixel‐level tumor detector. Therefore, we adopted an alternative method by padding background pixels to adjust H&E tiles that do not meet the resolution requirements. Classical data augmentation methods involve rotating and mirroring the original H&E tiles to generate more tiles. The morphological features of these augmented tiles remain unchanged compared to the original tiles, and they do not affect clinical diagnosis, making them suitable for model training. Additionally, we employed a recently popular pixel‐level masking method for data augmentation. This method involves randomly masking 1%‐5% of the pixels in the original H&E tile, introducing some artificial noise to simulate local image issues that do not affect the overall interpretation in the clinical diagnosis of H&E WSIs.^[^ ^22^ ^]^

Despite the satisfactory performance of HER2Net, there are still limitations. First, more clinical samples are needed to validate the reliability of HER2Net. Second, due to the constraints of Graphics Processing Unit (GPU) resources and sample size, the most advanced achievements in the field of deep learning have not been applied in this study. For instance, models based on Vision Transformer^[^ ^23^ ^]^ have not been attempted for tile‐level classifiers. The semantic segmentation model Segment Anything Model (SAM),^[^ ^24^ ^]^ which can be prompted, has also not been attempted for pixel‐level tumor detectors. In the future, more attention should be paid to the application of mature multimodal large language models on histopathological image data.

## 4 Experimental Section

### Study Participants

This study received approval from the Institutional Review Board of Southern Medical University (NFEC‐2023‐056). For the development of HER2Net, two pathological image datasets were utilized, namely the internal dataset from a single medical center, named Internal‐STAD, and the external dataset from multiple medical centers (Multi‐Center‐STAD). The selection criteria for this study included the following: 1) patients diagnosed with GC, who underwent primary gastrectomy at the respective hospitals within the period from January 1, 2012, to December 31, 2021; 2) patients possessing identified HER2 status; 3) accessibility to the clinical data and Hematoxylin and Eosin‐stained tumor slides. The criteria for exclusion encompassed the subsequent conditions: 1) patients who underwent preoperative treatments, for instance, neoadjuvant radiotherapy or chemotherapy; 2) patients whose clinical data was incomplete; 3) instances of inadequate slide scanning, such as out‐of‐focus slides or apparent tissue folds. The requirement for informed consent was exempted due to the fact that patients were not directly enlisted for this study.

### Slide Scanning and Annotations

Each patient from Internal‐STAD and the Multi‐Center‐STAD has at least one representative H&E tumor slide and its corresponding IHC slide. The slides were scanned using Motic EasyScanner at 4× magnification to obtain a TIFF format WSI file. Then, the Tag Image File Format (TIFF) format files were converted to Portable Network Graphics(PNG) format image files without loss using a computer vision open‐source library in Python to fit the deep learning framework. The annotation process consisted of two stages: first, two senior pathologists with at least five years of experience used a pathology image annotation tool to annotate and verify the tumor regions in each H&E WSI; then, the H&E tiles were further classified based on the IHC WSI for annotation. H&E tiles that cannot be accurately annotated due to reasons such as image blurriness, contamination, and insufficient clarity were documented and have not been used for model training.

### Interpretation of HER2 IHC and IHS Assays

IHC assesses the membranous immunostaining of neoplastic cells, utilizing a threshold of ≥10% immunoreactive tumor cells. A subsequent validation study confirmed the reproducibility of this scoring protocol among diverse pathologists. A score of 0, indicating membranous reactivity in <10% of neoplastic cells, or 1+, signifying faint membranous reactivity in at least 10% of neoplastic cells, is deemed to be HER2‐negative. Conversely, a score of 2+, representing weak to moderate membranous reactivity in at least 10% of neoplastic cells, is deemed equivocal, necessitating further examination via FISH or alternative ISH methods. The outcomes of FISH/ISH are presented as a ratio of the ERBB2 gene copy number to the count of centromeres on chromosome 17 (CEP17) within the nucleus, as observed in a minimum of 20 cancer cells (ERBB2:CEP17). Alternatively, FISH/ISH results can be represented as the mean ERBB2 copy number per cell. Cases demonstrating an IHC score of 3+ (indicating strong membranous reactivity in 10% or more cancer cells) or an IHC score of 2+ that are also FISH/ISH positive are classified as exhibiting HER2 high‐expression. HER2 IHC results that are either positive (3+) or negative (0 or 1+) do not necessitate further ISH testing.^[^ ^10^ ^]^

### Determination of HER2 Status

The HER2 IHC review was re‐conducted for this study to ensure consistency. The determination of the ground‐truth HER2 status from the Internal‐STAD and Multi‐Center‐STAD datasets was achieved through the utilization of HER2 IHC (HER2 IHC 2+ in conjunction with FISH) in histopathologic samples at their respective institutions (Figure S1, Supporting Information).

### WSI Processing

All H&E WSIs were cropped into non‐overlapping 512‐by‐512 resolution H&E tiles. H&E tiles with insufficient resolution after cropping were filled with white pixels considered as the background color to meet the 512‐by‐512 resolution. To improve the robustness and generalization ability of the model, data augmentation strategies were applied to the H&E tiles. Specifically, three strategies were used: 1) rotating the original H&E tile by 90 degrees, 180 degrees, and 270 degrees to obtain three new H&E tiles; 2) mirroring the original image horizontally and vertically to obtain two new H&E tiles; 3) randomly masking 1–5% of the pixels in the original H&E tile, which introduces some artificial noise to simulate the local image problems that do not affect the overall interpretation in clinical diagnosis of H&E WSI.^[^ ^22^ ^]^ The study calculated the saturation of each H&E tile and ultimately determined that tiles with a saturation less than or equal to ten were considered pure background tiles. Pure background tiles were not used for the subsequent training of tumor detector and HER2Net.

Similar data processing was applied to IHC WSI as did for H&E WSI, specifically cropping and background padding. Subsequently, it was manually ensured that the cropped images from the H&E WSI and IHC WSI of the same case were aligned on a one‐to‐one basis. Consequently, the annotation labels for the IHC tiles could also be applied to their corresponding H&E tiles. It is important to note that IHC tiles were used solely for data annotation in the tile‐level classifier of HER2Net and were not directly utilized for model training.

### Development of Pixel‐Level Tumor Detector

In clinical pathological diagnosis, the determination of HER2 status is only related to the histopathologic features of the tumor portion of the H&E WSI, and is unrelated to non‐tumor and background areas. Therefore, the study needs to train a tumor detector to identify the tumor regions in the H&E WSI, and non‐tumor and background regions will not be used for HER2Net training and inference. The method proposed in this paper required a quantitative calculation of the proportion of HER2 high‐expression regions. To meet this requirement, the study expected to achieve pixel‐level precision of tumor boundaries by performing a binary classification of whether each pixel in the H&E WSI is a tumor. Tumor detectors were trained for pixel‐level tumor detection in H&E WSI using three classic deep learning models in the field of image semantic segmentation:^[^ ^21^ ^]^ U‐Net,^[^ ^25^ ^]^ SegNet,^[^ ^26^ ^]^ and DeepLabv3+.^[^ ^27^ ^]^ The study used the Adam^[^ ^28^ ^]^ optimizer on two NVIDIA RTX A6000 48G GPUs for parallel training, with a starting learning rate of 10^−5^, weight decay of 0.001, and a mini batch size of 32. The loss function was cross‐entropy, and the evaluation metric was MIoU. Table S1 (Supporting Information) records the performance of the three models on both the Internal‐STAD test set and the Multi‐Center‐STAD test set. Based on the results, SegNet achieved the best performance with an MIoU of 0.8606 on the Internal‐STAD test set and an MIoU of 0.8207 on the Multi‐Center‐STAD test set. Therefore, SegNet was selected as the final tumor detector. Examples of heatmap of tumor regions annotated by pathology experts and predicted by tumor detector are shown in Figure S3 (Supporting Information) respectively.

### Non‐Tumor Tiles Filter

The study defines non‐tumor tiles as tiles where the percentage of tumor pixels predicted by the tumor detector is less than or equal to 10%, or the saturation is less than 10. Non‐tumor tiles have not been used for training and inference of HER2Net.

### Development of HER2Net

*Tile‐Level Classifier*. The workflow of HER2Net is depicted in Figure 2. The HER2Net first comprises a tile‐level classifier, which performs binary classification on H&E tiles. When classifying the H&E tile, it was defined that if the high‐expression area of the corresponding IHC tile is greater than or equal to 50% of the tumor area, it is classified as strong. Conversely, if the high‐expression area of the corresponding IHC tile is <50% of the tumor area, it is classified as weak. During the training process, the study adopted a strategy of fivefold cross‐validation and stratified sampling on WSI‐level, resulting in five tile‐level sub‐classifiers. Fivefold cross‐validation involved dividing the internal training set evenly into five subsets, with four subsets for training and the remaining subset for validation. The training was terminated when the tile‐level sub‐classifier's performance has no longer improvement on the validation set. Stratified sampling is utilized to ensure the proportion of HER2 positive samples within each fold of the fivefold cross‐validation is as consistent as possible, with the aim of enhancing the robustness of the model. During the process of model inference, each sub‐classifier makes a prediction for the classification of the H&E tile. Therefore, a single H&E tile can yield five prediction results from five sub‐classifiers. Tile‐level classifier was trained using the ResNet series models^[^ ^29^ ^]^ and DenseNet series models,^[^ ^30^ ^]^ including ResNet152, ResNet101, ResNet50, ResNet34, ResNet18 and DenseNet121. The Adam optimizer was used on two NVIDIA RTX A6000 48G GPUs for parallel training, with a starting learning rate of 10^−5^, weight decay of 0.001, and a mini batch size of 64. The loss function is cross‐entropy, and two evaluation metrics AUROC and sensitivity were used for comprehensive evaluation. Table S2 (Supporting Information) shows the best average performance of those models in multiple stratified fivefold cross‐validation experiments on the internal training set. According to the results, ResNet50 achieved the best average performance with an AUROC of 0.9379. Therefore, ResNet50 was selected as the final tile‐level classifier.

*Integrated Classifier*. HER2Net second included an integrated classifier to learn the final classification prediction by combining the classification results obtained from the previous five tile‐level sub‐classifiers. For training the integrated classifier, three linear classification models were tested including Logistic Regression (LR),^[^ ^31^ ^]^ Decision Tree (DT),^[^ ^32^ ^]^ and Support Vector Machine (SVM),^[^ ^33^ ^]^ as well as five ensemble learning models^[^ ^34^ ^]^ including Random Forest (RF), Adaptive Boosting (AdaBoost), Gradient Boosting Decision Tree (GBDT), Extreme Gradient Boosting (XGBoost), and Soft Voting (SV). The study trained the above eight models using the grid search to find the best combinations of model parameters on a single CPU. Two evaluation metrics AUROC and sensitivity were used for comprehensive evaluation. All test results on the MultiCenter‐STAD test set are recorded in Table S3 (Supporting Information). According to the results, the AUROC of all models on Multi‐Center‐STAD test set ranges from 0.9769 to 0.9898, with little difference. However, the sensitivity of RF is 0.9373, significantly higher than other models. Considering that classifying as strong is a minority class sample and has a significant impact on HER2 status determination compared to classifying as weak, the study has chosen RF as the final integrated classifier.

*High‐Expression Percent Calculator*. HER2Net third included a high‐expression percent calculator, which was used to calculate the proportion of HER2 high‐expression regions in H&E WSI. For a given H&E WSI, it is assumed to be divided into a set of non‐overlapping H&E tiles, and those H&E tiles containing tumors are selected using a tumor detector. Moreover, the pixel‐level tumor detector can also provide the number of tumor pixels for each H&E tile. The classification of each H&E tile is obtained using an integrated classifier. Afterward, it was defined that all tumor pixels in H&E tiles classified as strong are considered as HER2 high‐expression pixels, while all tumor pixels in H&E tiles classified as weak are considered as HER2 non‐high‐expression pixels. By accumulating the high‐expression and non‐high‐expression pixels of each H&E tile, the proportion of high‐expression P in the H&E can be calculated WSI using the following formula:

(1) $$P\;=\frac{\sum_{i=1}^{N}K_{i}}{\sum_{i=1}^{N}K_{i}+\sum_{j=1}^{M}L_{j}}\;$$

where N and M represent the number of strong H&E tiles and weak H&E tiles, respectively, in one H&E WSI. K_i_ represents the number of high‐expression pixels in the i‐th strong tile, while L_j_ represents the number of non‐high‐expression pixels in the j‐th weak tile. When P is greater than or equal to 10% (the threshold used in clinical immunohistochemistry assessment), the H&E WSI will be determined as HER2 positive. Conversely, when P is less than 10%, the H&E WSI will be determined as HER2 negative.

All models involved in the experiments of this paper, along with their corresponding literature and code links, are comprehensively documented in Table S5 (Supporting Information). The mathematical principles of the models are thoroughly explained in their respective literature. The code implementations provided in the links are open‐source and freely available for use.

## Conflict of Interest

The authors declare no conflict of interest.

## Author Contributions

Y.L., X.C., and S.H. contributed equally to this work. Z.L., L.Y.H., C.X.H., and H.S.P. designed the study. Z.X.H., X.H., and Z.Y.F. collected the samples and acquired the image data. Z.L., C.X.H., W.X.J., and Z.D.H. provided the clinical and pathological data of multiple medical centers. H.S.P. and L.Y.H. performed the machine learning. Z.L., C.B., W.X.J., Z.X.F., and Z.H.M. conducted the labeling. H.S.P, L.Y.H., and Z.X.H. did the statistical analyses. All authors vouch for the data, analyses, and interpretations. Z.L., L.Y.H., H.S.P., and C.X.H. wrote the first draft of the manuscript, and all authors reviewed, contributed to, and approved the manuscript.

## Supporting information



Supporting Information

## Data Availability

The data that support the findings of this study are available in the supplementary material of this article.
