# Supplementary material for: Artificial Intelligence for Predicting HER2 Status of Gastric Cancer Based on Whole‐Slide Histopathology Images: A Retrospective Multicenter Study
Source: Adv Sci (Weinh). 2025 Jan 10;12(10):2408451. doi: 10.1002/advs.202408451 (PMC11904990; doi:10.1002/advs.202408451)
Supplement: Supplementary file 1 — Supporting Information [file ADVS-12-2408451-s001.docx]

**Supplementary figures**


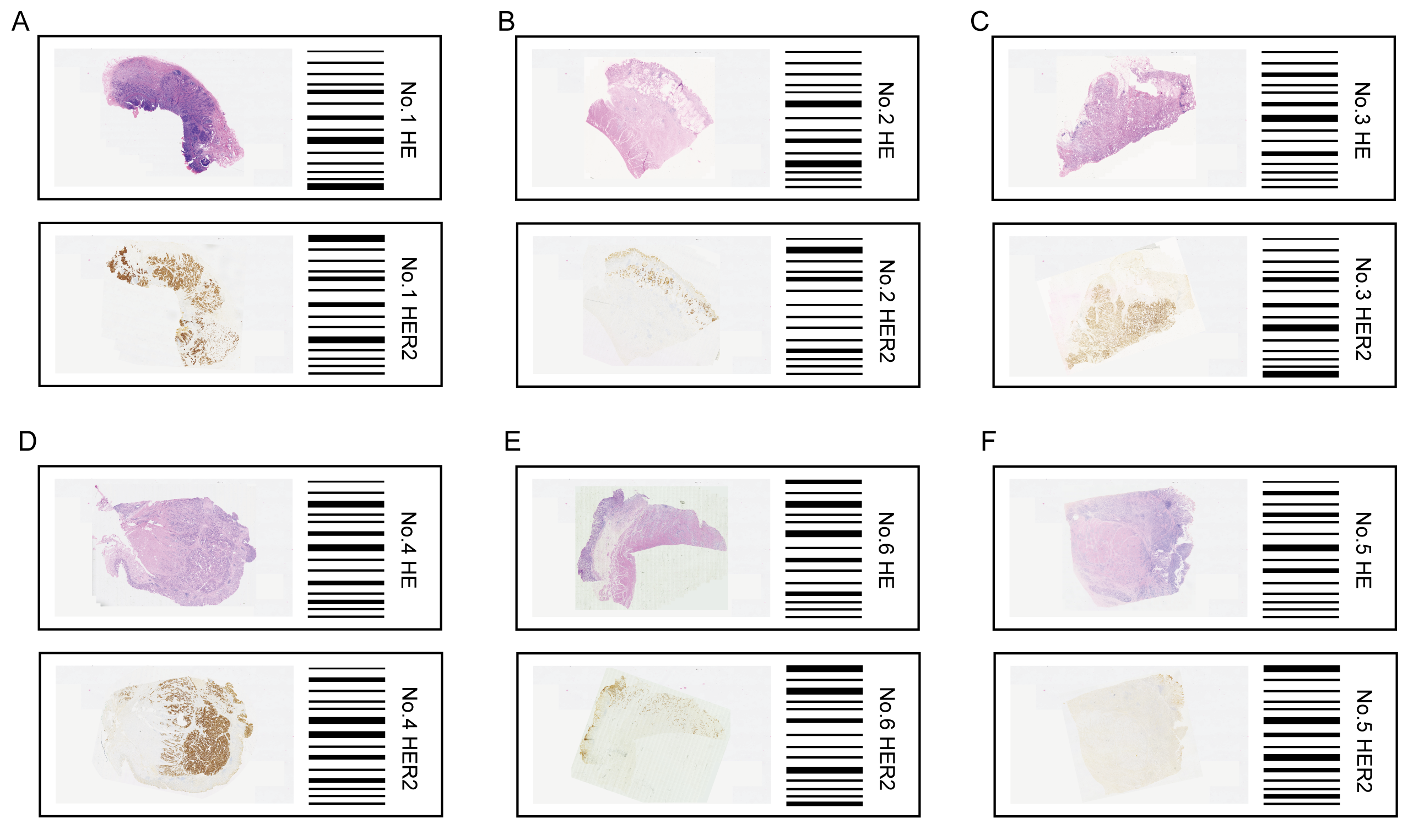


**Supplementary Figure S1**. **Re-conducted of HER2 status using IHC in histopathologic samples. (A-E)** The H&E WSI of HER2-positive patients, along with their corresponding IHC WSI. (**F)** The H&E WSI and corresponding IHC WSI of HER2-negative patients.


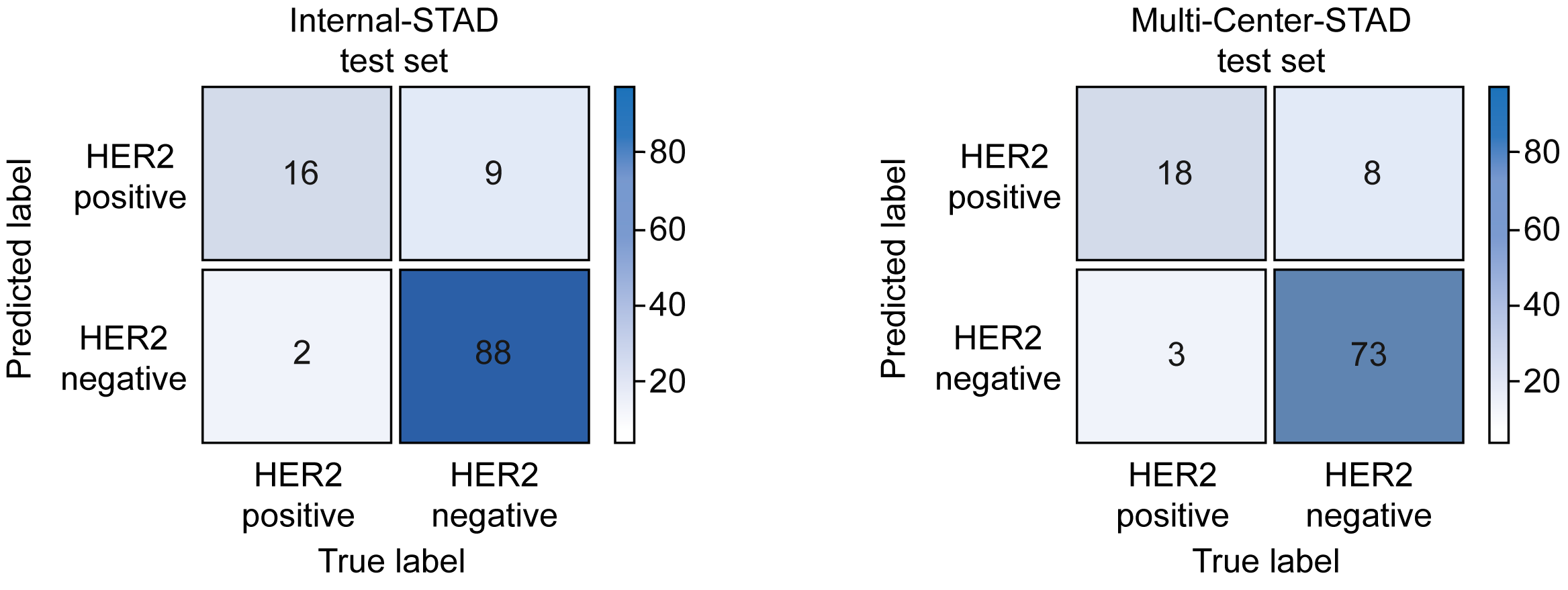


**Supplementary Figure S2. Confusion matrix based on the ground-truth HER2 status of all patients and predictions by HER2Net on Internal-STAD test set and Multi-Center-STAD test set, respectively.** Frequencies are displayed on a color gradient scale.


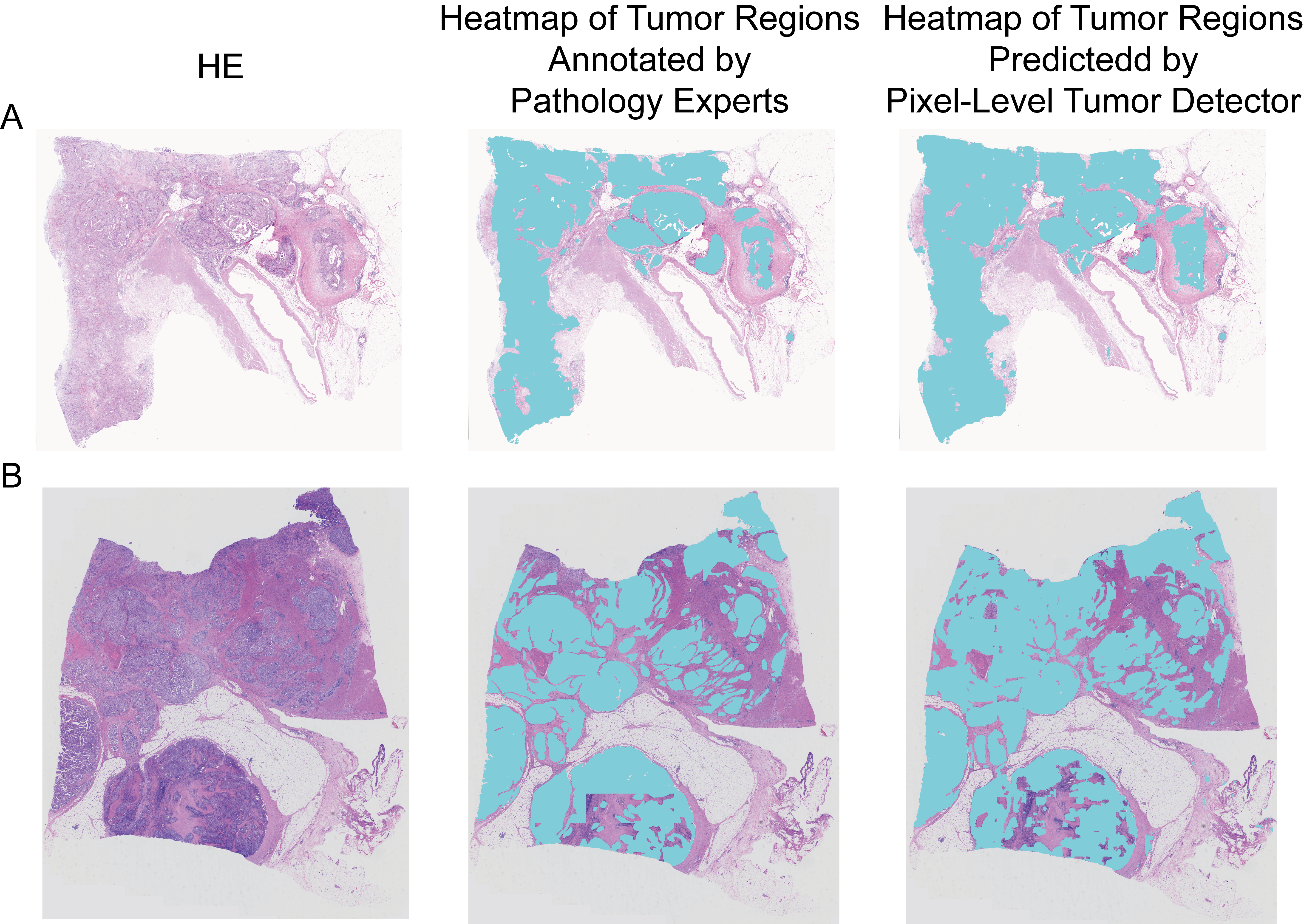


**Supplementary Figure S3. Examples of heatmap of tumor regions annotated by pathology experts (displayed in the second column) and predicted by tumor detector (displayed in the thirdcolumn) respectively.**

**Supplementary Tables**

**Supplementary Table S1. Comparison of different models of tumor detector on the test set.**

| Model | Internal-STAD test set | | | | Multi-Center-STAD test set | | | |
| --- | --- | --- | --- | --- | --- | --- | --- | --- |
|  | PA | MPA | MIoU | FWIoU | PA | MPA | MIoU | FWIoU |
| U-Net | 0.8742 | 0.8367 | 0.8166 | 0.8313 | 0.8317 | 0.8274 | 0.7874 | 0.8156 |
| SegNet | 0.9387 | 0.9285 | **0.8606** | 0.8865 | 0.9150 | 0.9069 | **0.8207** | 0.8463 |
| DeepLabv3+ | 0.9080 | 0.8655 | 0.8304 | 0.8499 | 0.8764 | 0.8698 | 0.8076 | 0.8249 |

**Note:** PA, Pixel Accuracy; MP, Mean Pixel Accuracy; MIoU, Mean Intersection over Union; FWIoU, Frequency Weighted Intersection over Union.

**Supplementary Table S2. Comparison of different models of tile-level classifier on the internal training set by stratified 5-fold cross validation.**

| Model | 5-Fold Averaged | | | | | | | |
| --- | --- | --- | --- | --- | --- | --- | --- | --- |
|  | Precision | | Recall | | F1-score | | Accuracy | AUROC |
|  | Weak | Strong | Weak (Specificity) | Strong (Sensitivity) | Weak | Strong |  |  |
| ResNet152 | 0.9876 | 0.8151 | 0.9779 | 0.8870 | 0.9827 | 0.8490 | 0.9690 | 0.9325 |
| ResNet101 | 0.9877 | 0.8304 | 0.9802 | 0.8881 | 0.9840 | 0.8582 | 0.9712 | 0.9342 |
| ResNet50 | 0.9883 | 0.8471 | 0.9825 | 0.8933 | 0.9854 | 0.8695 | 0.9737 | 0.9379 |
| ResNet34 | 0.9882 | 0.8338 | 0.9805 | 0.8925 | 0.9843 | 0.8616 | 0.9719 | 0.9365 |
| ResNet18 | 0.9868 | 0.8514 | 0.9528 | 0.8793 | 0.9832 | 0.8509 | 0.9698 | 0.9294 |
| DenseNet121 | 0.9856 | 0.8282 | 0.9803 | 0.8682 | 0.9829 | 0.8474 | 0.9693 | 0.9243 |

**Note:** Strong, the high expression area of the corresponding IHC tile is greater than or equal to 50% of the tumor area; Weak, the high expression area of the corresponding IHC tile is less than 50% of the tumor area; Precision, the ratio of correctly predicted observations to the total predicted observations; Recall, the ratio of correctly predicted observations to the all observations in actual class; Specificity, true positive rate; Sensitivity, true negative rate; f1-score, ; AUROC, Area under the Receiver Operating Characteristic Curve.

**Supplementary Table S3. Comparison of different models of integrated classifier on the MultiCenter-STAD test set.**

| Model | Multi-Center-STAD test set | | | | | | | |
| --- | --- | --- | --- | --- | --- | --- | --- | --- |
|  | Precision | | Recall | | F1-score | | Accuracy | AUROC |
|  | Weak | Strong | Weak (Specificity) | Strong (Sensitivity) | Weak | Strong |  |  |
| SV | 0.9804 | 0.8819 | 0.9897 | 0.7945 | 0.9850 | 0.8359 | 0.9826 | 0.9883 |
| LR | 0.9784 | 0.8923 | 0.9910 | 0.7726 | 0.9846 | 0.8281 | 0.9718 | 0.9876 |
| DT | 0.9823 | 0.8789 | 0.9892 | 0.8149 | 0.9857 | 0.8457 | 0.9739 | 0.9861 |
| SVM | 0.9774 | 0.9064 | 0.9924 | 0.7624 | 0.9849 | 0.8282 | 0.9722 | 0.9898 |
| RF | 0.9938 | 0.7374 | 0.9678 | **0.9373** | 0.9806 | 0.8254 | 0.9651 | 0.9769 |
| AdaBoost | 0.9846 | 0.7787 | 0.9770 | 0.8411 | 0.9808 | 0.8087 | 0.9650 | 0.9873 |
| GBDT | 0.9873 | 0.8407 | 0.9848 | 0.8309 | 0.9843 | 0.8358 | 0.9713 | 0.9836 |
| XGBoost | 0.9801 | 0.8772 | 0.9893 | 0.7915 | 0.9847 | 0.8322 | 0.9719 | 0.9878 |

Note: SV, Soft Voting; LR, Logistic Regression; DT, Decision Tree; SVM, Support Vector Machine; RF, Random Forest; AdaBoost, Adaptive Boosting; GBDT, Gradient Boosting Decision Tree; XGBoost, Extreme Gradient Boosting.

**Supplementary Table S4. Best performance HER2Net2 achieved in predicting the HER2 status.**

| Model | Internal-STAD test set | | | | | Multi-Center-STAD test set | | | | |
| --- | --- | --- | --- | --- | --- | --- | --- | --- | --- | --- |
|  | Precision | | Recall | | Accuracy | Precision | | Recall | | Accuracy |
|  | Negative | Positive | Negative (Specificity) | Positive (Sensitivity) |  | Negative | Positive | Negative (Specificity) | Positive (Sensitivity) |  |
| SegNet+ResNet50+RF | 0.9778 | 0.6400 | 0.9072 | 0.8889 | 0.9043 | 0.9605 | 0.6923 | 0.9012 | 0.8571 | 0.8922 |

**Note:** Negative, HER2 negative; Positive, HER2 positive.

**Supplementary Table S5. Models, references and code link.**

| Model | Task | Trained as | Reference Number | Code link |
| --- | --- | --- | --- | --- |
| U-Net | Image semantic segmentation | Pixel-level tumor detector | [23] | https://lmb.informatik.uni-freiburg.de/people/ronneber/u-net/ |
| SegNet |  |  | [24] | https://arxiv.org/abs/1511.00561 |
| DeepLabv3+ |  |  | [25] | https://github.com/tensorflow/models/tree/master/research/deeplab |
| ResNet series models | Image classification | Tile-level classifier | [27] | Integrated in torchvision of PyTorch, refer to the link: https://pytorch.org/vision/stable/models.html#classification |
| DenseNet series models |  |  | [28] |  |
| LR | Linear classification | Integrated classifier | [29] | Integrated in sklearn.linear_model, refer to the link: https://scikit-learn.org/0.21/modules/classes.html#module-sklearn.linear_model |
| DT |  |  | [30] | Integrated in sklearn.tree, refer to the link: https://scikit-learn.org/0.21/modules/classes.html#module-sklearn.svm |
| SVM |  |  | [31] | Integrated in sklearn.svm, refer to the link: https://scikit-learn.org/0.21/modules/classes.html#module-sklearn.tree |
| SV | Ensemble learning for classification |  | [32] | Integrated in sklearn.ensemble, refer to the link: https://scikit-learn.org/0.21/modules/classes.html#module-sklearn.ensemble |
| RF |  |  |  |  |
| AdaBoost |  |  |  |  |
| GBDT |  |  |  |  |
| XGBoost |  |  |  |  |
